# Tumor-Related Methylated Cell-Free DNA and Circulating Tumor Cells in Melanoma

**DOI:** 10.3389/fmolb.2015.00076

**Published:** 2016-01-08

**Authors:** Francesca Salvianti, Claudio Orlando, Daniela Massi, Vincenzo De Giorgi, Marta Grazzini, Mario Pazzagli, Pamela Pinzani

**Affiliations:** ^1^Department of Clinical, Experimental and Biomedical Sciences, University of FlorenceFlorence, Italy; ^2^Division of Pathology, Department of Surgery and Translational Medicine, University of FlorenceFlorence, Italy; ^3^Division of Dermatology, Department of Surgery and Traslational Medicine, University of FlorenceFlorence, Italy

**Keywords:** circulating tumor cells, cell-free DNA, epigenetic marker, melanoma, tumor marker

## Abstract

Solid tumor release into the circulation cell-free DNA (cfDNA) and circulating tumor cells (CTCs) which represent promising biomarkers for cancer diagnosis. Circulating tumor DNA may be studied in plasma from cancer patients by detecting tumor specific alterations, such as genetic or epigenetic modifications. Ras association domain family 1 isoform A (*RASSF1A*) is a tumor suppressor gene silenced by promoter hypermethylation in a variety of human cancers including melanoma. The aim of the present study was to assess the diagnostic performance of a tumor-related methylated cfDNA marker in melanoma patients and to compare this parameter with the presence of CTCs. *RASSF1A* promoter methylation was quantified in cfDNA by qPCR in a consecutive series of 84 melanoma patients and 68 healthy controls. In a subset of 68 cases, the presence of CTCs was assessed by a filtration method (Isolation by Size of Epithelial Tumor Cells, ISET) as well as by an indirect method based on the detection of tyrosinase mRNA by RT-qPCR. The distribution of *RASSF1A* methylated cfDNA was investigated in cases and controls and the predictive capability of this parameter was assessed by means of the area under the ROC curve (AUC). The percentage of cases with methylated *RASSF1A* promoter in cfDNA was significantly higher in each class of melanoma patients (*in situ*, invasive and metastatic) than in healthy subjects (Pearson chi-squared test, *p* < 0.001). The concentration of *RASSF1A* methylated cfDNA in the subjects with a detectable quantity of methylated alleles was significantly higher in melanoma patients than in controls. The biomarker showed a good predictive capability (in terms of AUC) in discriminating between melanoma patients and healthy controls. This epigenetic marker associated to cfDNA did not show a significant correlation with the presence of CTCs, but, when the two parameters are jointly considered, we obtain a higher sensitivity of the detection of positive cases in invasive and metastatic melanomas. Our data suggest that cell-free tumor DNA and CTCs represent two complementary aspects of the liquid biopsy which may improve the diagnosis and the clinical management of melanoma patients.

## Introduction

Cell-free DNA (cfDNA) and circulating tumor cells (CTCs), released into the bloodstream by solid tumors, are considered real time liquid biopsies in cancer patients reflecting the disease complexity at any stage of cancer progression. The liquid biopsy represents a surrogate material for the molecular characterization of solid cancers (Hodgson et al., 2010) which is particularly valuable after the resection of primary tumor and in the metastatic patients when multiple distinct tumor masses are simultaneously present. In fact, in the advanced stages when the tumor has acquired genetic, epigenetic and expression features which may be very different from those of the primary tissue, the liquid biopsy should represent a picture of the current molecular state of the disease collecting cellular and molecular markers from all the tumor sites in the organism.

CTCs and cfDNA are usually investigated separately and only few studies assayed both CTCs and cfDNA focusing on the correlation between the two parameters (Alix-Panabières et al., 2012).

Total cfDNA concentration, higher in cancer patients than healthy individuals (Fleischhacker and Schmidt, 2007), has been proposed as a cancer marker, but it has shown limited sensitivity and specificity (Jung et al., 2010).

On the other hand the identification tumor cfDNA, i.e., the fraction of cfDNA deriving from the tumor, may help reaching a higher diagnostic specificity. This task can be accomplished by detecting tumor specific alterations, such as epigenetic modifications among which aberrant DNA methylation in the promoter region of tumor suppressor genes (Board et al., 2008) plays a role in cancer progression and maintenance. Aberrant cfDNA methylation has been described in most cancer types and is being investigated for clinical applications (Warton and Samimi, 2015). Methylation is a promising target for biomarker development due to the stability of CpG island methylation changes by comparison with the high variability of cancer mutation profiles within a specific cancer (Warton and Samimi, 2015). In addition, methylated tumor cfDNA may be a more sensitive marker for early-stage diseases as DNA methylation is often an early event in carcinogenesis (Diaz and Bardelli, 2014).

*RASSF1A* (Ras association domain family 1 isoform A) is a tumor suppressor gene, whose inactivation, mainly achieved by promoter hypermethylation, is involved in the development of many cancers (Donninger et al., 2007, 2015). So far the role of *RASSF1A* methylation as a biomarker in cfDNA has been investigated, mostly in combination with other parameters, in a variety of tumors such as breast cancer (Papadopoulou et al., 2006; Skvortsova et al., 2006; Agostini et al., 2012), prostate cancer (Papadopoulou et al., 2006), lung cancer (Begum et al., 2011; Ponomaryova et al., 2013), ovarian carcinoma (Bondurant et al., 2011; Liggett et al., 2011; Zhang et al., 2013), gastric cancer (Balgkouranidou et al., 2015), testicular germ cell cancer (Ellinger et al., 2009), nasopharyngeal carcinoma (Wong et al., 2004), renal cell carcinoma (de Martino et al., 2012), bladder cancer (Hauser et al., 2013), and melanoma (Hoon et al., 2004; Koyanagi et al., 2006; Salvianti et al., 2012). In this neoplasia *RASSF1A* promoter methylation has a frequency of 55% (Spugnardi et al., 2003). Methylation of *RASSF1A* increases significantly with advancing clinical stage, suggesting that the inactivation of this gene is associated with tumor progression (Tanemura et al., 2009). *RASSF1A* promoter hypermethylation has been detected in cfDNA from melanoma patients (Hoon et al., 2000; Marini et al., 2006) in association to a worse response to therapy and reduced overall survival (Mori et al., 2005; Koyanagi et al., 2006).

CTCs have been detected and characterized by means of different methods in a variety of tumors, but their identification as extremely rare cells in blood is challenging. In melanoma, RT-qPCR is one of the most widely used methods for the indirect detection of CTCs (Rodic et al., 2014), demonstrating high sensitivity through the detection of marker RNA expression such as tyrosinase mRNA coding for a melanocyte-specific enzyme. Other methods used to investigate the presence of CTCs in melanoma are able to maintain cell integrity separating CTCs on the basis of their larger size with respect to blood cells (Rodic et al., 2014). Studies on melanoma patients by different technical approaches showed CTC detection rates ranging from 14 to 49% in stage III and from 40 to 72% in stage IV melanomas (Khoja et al., 2015).

The aim of the present paper was to investigate the role of circulating methylated *RASSF1A* as a non-invasive marker in melanoma patients and to compare *RASSF1A* methylation status in cfDNA with the presence of CTCs identified by two different methods (isolation of CTCs by size and quantification of tyrosinase mRNA by RT-qPCR).

## Materials and methods

### Patients

Consecutive patients affected by primary and metastatic cutaneous melanoma (*n* = 84) were enrolled at diagnosis at the Department of Dermatological Sciences of the University of Florence. The cohort included 37 females and 47 males with median age of 62 years (range 23–94 years). Patients were affected by in situ (*n* = 14, 9 males and 5 females, median age 60 years, range 39–80), invasive (*n* = 60, 33 males and 27 females, median age 65 years, range 23–88), and metastatic melanoma (*n* = 10, 5 males and 5 females, median age 50 years, range 28–94). As a control population 68 healthy subjects were enrolled: 32 males and 36 females, median age 59 years (range 25–80 years). The clinicopathological characteristics of melanoma cases are reported in Table 1.

**Table 1:** **Clinicopathological characteristics of melanoma cases**.

**Parameter**	**Number of cases**	**Percent of cases**
Total	84	100%
**LOCATION**		
Head and Neck	6	7.1%
Limbs	24	28.6%
Chest	46	54.8%
Acral	5	6.0%
Genital	2	2.4%
Missing	1	
**THICKNESS**		
*In situ*	14	16.6%
≤ 1 mm	39	46.4%
1–2 mm	14	16.6%
2–4 mm	9	10.7%
>4 mm	3	3.6%
Missing	5	
**CLARK LEVEL**		
I	15	17.9%
II	14	16.7%
III	22	26.2%
IV	28	33.3%
Missing	5	
**ULCERATION**		
Absent	67	79.8%
Present	12	14.3%
Missing	5	
**SENTINEL LYMPH NODE**		
Negative	18	21.4%
Positive	3	3.6%
Not done	63	75.0%
**STAGE OF DISEASE**		
0	13	15.5%
1	46	54.8%
2	7	8.3%
3	6	7.1%
4	12	14.3%

The research protocol was approved by the institutional review board of the University of Florence and all the patients signed an informed consent.

### Sample collection

Blood samples (5 ml) were collected in EDTA tubes during the dermatologic examination and before surgery, prior to any treatment.

Plasma was separated within 3 h from blood draw by two centrifugation steps at 4°C for 10 min at 1600 rcf and 14000 rcf, respectively. Plasma aliquots (505 μl) were stored at −80°C. DNA was extracted from 500 μl of plasma by the QIAamp DSP Virus Kit (QIAgen, Germany) according to the manufacturer's instructions. cfDNA extraction and subsequent molecular analyses were performed consecutively during patients' and controls' enrolment.

### Quantification of the methylated form of *RASSF1A* promoter in cfDNA

The methylated form of *RASSF1A* promoter was quantified in plasma by a qPCR assay according to an already described protocol (Chan et al., 2006), after digestion of unmethylated DNA by the methylation-sensitive enzyme Bsh1236I (Fermentas, Canada) in a reaction volume of 25 μl at 37°C for 16 h. Subsequently, 5 μl of enzyme-treated DNA underwent a qPCR assay for *RASSF1A* promoter, in a final volume of 25 μl, according to the protocol by Chan et al. (2006). As a control for the enzymatic reaction we used a qPCR assay targeting an unmethylated sequence of the *ACTB* promoter: the absence of amplification signals for *ACTB* indicated a complete digestion of the sample. A reference curve obtained by serial dilutions of genomic DNA was used to quantify the methylated alleles. Results were expressed as genomic equivalents (GE, each corresponding to 6.6 pg DNA) per ml plasma (GE/ml pl).

### Quantification of total cfDNA

The quantity of the cfDNA circulating in plasma was evaluated by a qPCR assay targeting the human gene *APP* (Amyloid Precursor Protein, chr. 21q21.2, accession NM_000484), as already reported (Pinzani et al., 2010a). The cfDNA concentration was obtained by interpolation on an external reference curve of genomic DNA ranging from 10 to 10^5^ pg/reaction. The results have been used to calculate the percentage of tumor cfDNA applying the following formula:

$$\begin{array}{l} \%\text{tumor cfDNA }=\;[\text{RASSF1A methylated promoter}\\ \;(\text{GE/ml plasma})/\text{APP }(\text{GE/ml plasma})]\times 100. \end{array}$$

### Detection of circulating tumor cells (CTCs)

The presence of CTCs was assessed in a subgroup of 68 patients by the following two different methods:

#### Isolation by size of epithelial tumor cells (ISET)

Patients' blood (10 ml) was collected in EDTA tubes and processed within 4 h. ISET was performed by means of an ISET Device (Rarecells, France) as previously described (De Giorgi et al., 2010). Briefly, the separation of CTCs is obtained by filtration of blood through a membrane with 8 μm pores after lysis of red blood cells. Filters are stained with hematoxylin and eosin. Samples with the presence of at least one CTC were considered positive.

#### RT-qPCR for tyrosinase mRNA

The presence of CTCs was determined by an indirect method based on the detection by RT-qPCR of the mRNA for tyrosinase, an enzyme which is involved in the biosysnthesis of the melanin, thus specifically expressed by melanocytes and melanoma cells.

The protocol has been already reported (Pinzani et al., 2010b). Briefly, total RNA from whole blood was isolated by the PAXgene Blood RNA kit (PreAnalytiX, Switzerland), according to the manufacturer's instructions. RNA (500 ng) was reverse-transcribed using Taqman Reverse Transcription Reagents (Applied Biosystems, USA). To detect the tyrosinase transcript a Taqman Gene Expression Assay (ID: Hs00165976_m1, Applied Biosystems) was used. To calculate the expression of tyrosinase mRNA in each sample, we referred to an external reference curve generated by spiking a given number of cells of the melanoma cell line SKMEL28 into the blood from healthy donors (range 4000–0.4 cells/ml blood). Based on a previous study on uveal melanoma patients (Pinzani et al., 2010b) we established a cutoff for tyrosinase expression of 0.08 SKMEL28 cell equivalents/ml blood: all the samples showing a tyrosinase expression level above this cut-off value were considered positive for the presence of CTCs.

### Statistical analysis

Statistical differences among qualitative results were assessed by the Fisher's exact test or Pearson chi-squared test. Differences in quantities of *RASSF1A* methylated cfDNA among distinct classes of patients were assessed by the Mann–Whitney test. *P*-values lower than 0.05 were considered significant.

We investigated the predictive capability (i.e., diagnostic performance) of each marker by means of the area under the ROC curve (AUC).

Statistical analysis was carried out using the IBM SPSS Statistics software package, version 20.0 (IBM, NY, USA).

## Results

### *RASSF1A* promoter methylation in cfDNA

The methylated form of the *RASSF1A* promoter was quantified in cfDNA from melanoma patients and control subjects.

*RASSF1A* methylated alleles were detectable in 7/68 (10%) healthy subjects and in 39/84 (46%) melanoma patients with a significant association between the presence of methylated alleles in cfDNA and the subjects' category (Fisher's exact test, *p* < 0.001).

The percentage of cases with methylated *RASSF1A* promoter in cfDNA was higher respectively in *in situ* (8/14, 57%), invasive (24/60, 40%), and metastatic (7/10, 70%) melanoma patients than in controls (Pearson chi-squared test, *p* < 0.001).

We did not find any significant relationship between *RASSF1A* promoter methylation and the main clinicopathological parameters such as Breslow thickness, Clark level, histotype, and tumor site.

Quantitative data are reported in Table 2.

**Table 2:** **Quantitative data relative to *RASSF1A* promoter methylation**.

	**Controls**	**All melanoma patients (*in situ* +invasive +metastatic)**	***In situ***	**Invasive**	**Metastatic**
*RASSF1A* methylated cfDNA (GE/ml pl) median (range)	2.80 (0.89-4.01)	12.49 (1.20-208.68)	8.85 (2.96-21.06)	12.99 (1.20-71.40)	32.85 (7.71-208.68)
Total cfDNA (GE/ml pl)	848 (150-7195)	2363 (135-31600)	2690 (882-7329)	2316 (135-31600)	2402 (689-19017)
Percentage of tumor cfDNA median (Range)	0.21% (0.02-1.64%)	0.46% (0.02-11.55%)	0.37% (0.07-2.39%)	0.43% (0.02-11.55%)	0.80% (0.12-8.84%)

The concentration of *RASSF1A* methylated cfDNA in the subjects with a detectable quantity of methylated alleles was significantly higher in melanoma patients than in controls, *p* = 0.001.

**Figure 1:**
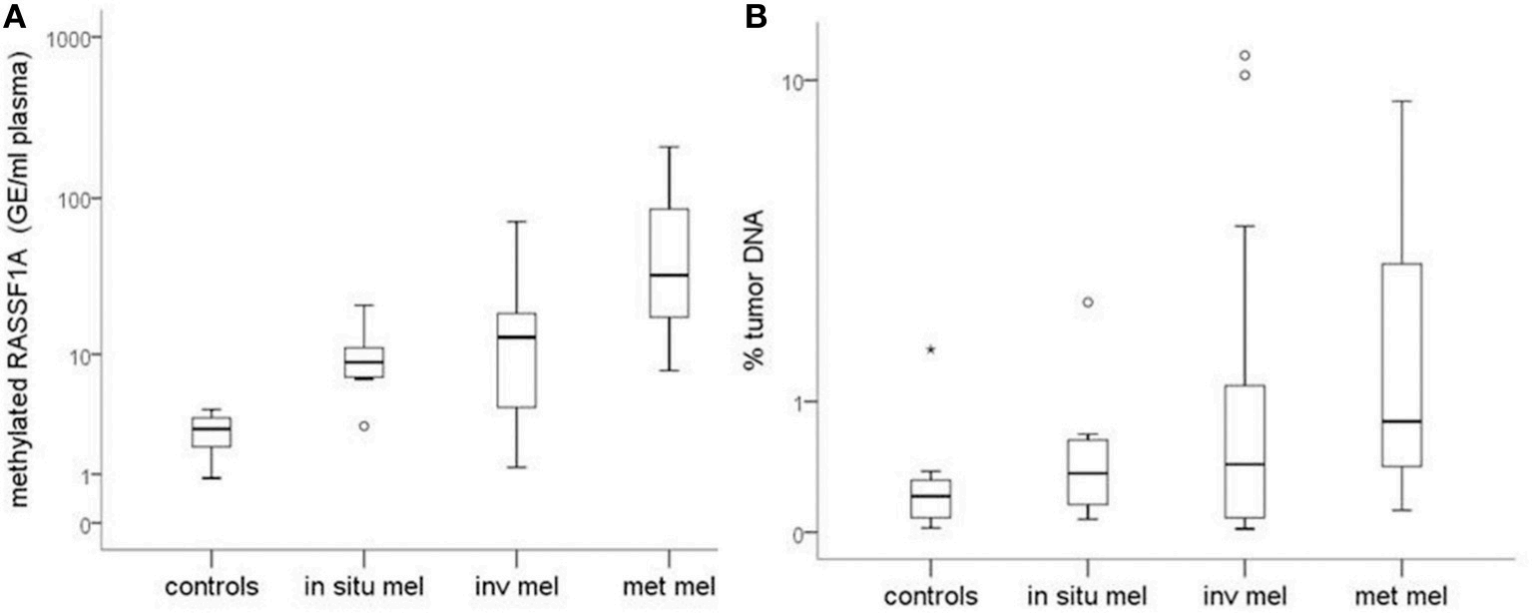
**(A)** Quantity of *RASSF1A* methylated cfDNA in different classes of melanoma patients and control subjects. **(B)** Percentage of tumor DNA circulating in the plasma of different classes of melanoma patients and control subjects. Box plots represent the median values and interquartile range of *RASSF1A* methylated cfDNA. mel, melanoma; inv, invasive; met, metastatic.

Figure 1A reports the median values and range of methylated *RASSF1A* in cfDNA for subjects with detectable levels of the marker. Control subjects show lower levels of circulating methylated *RASSF1A* promoter than patients affected by *in situ* (*p* = 0.004), invasive (*p* = 0.004) and metastatic melanoma (*p* = 0.002). Metastatic patients have higher concentrations of methylated *RASSF1A* alleles in plasma than patients with *in situ* (*p* = 0.011) and invasive melanoma (*p* = 0.021).

**Figure 2:**
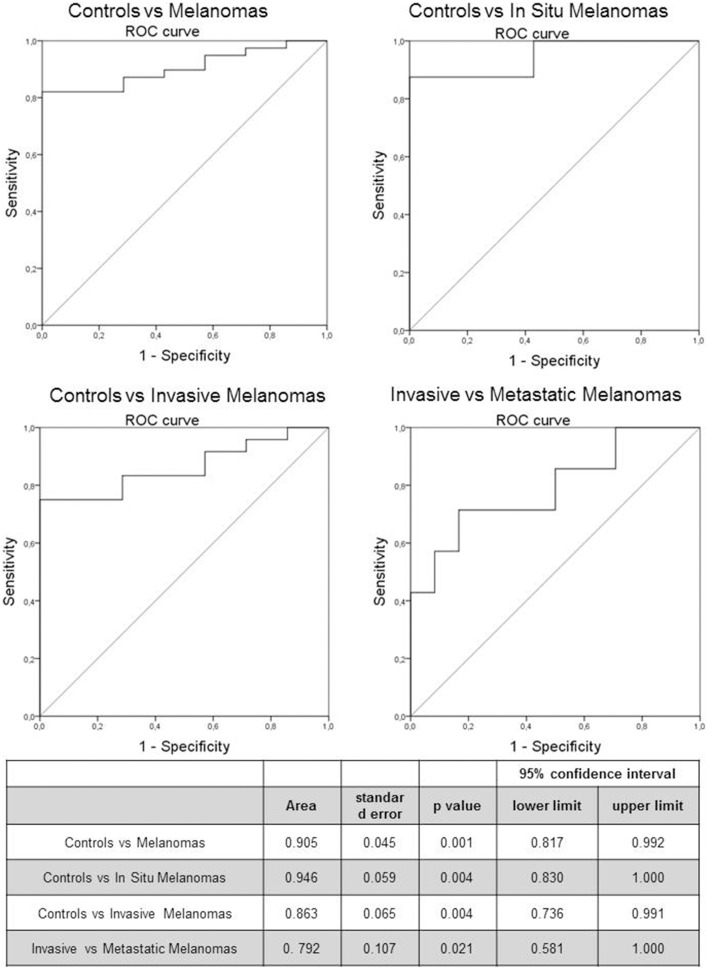
**Receiver Operating Characteristic (ROC) curve of *RASSF1A* methylated cfDNA in melanoma patients and control subjects**. The table reports the parameters of the ROC curve.

The predictive capability (i.e., diagnostic performance) of *RASSF1A* promoter methylation in plasma as a biomarker in melanoma was investigated by means of the area under the ROC curve. A good predictive capability was observed with an AUC of 0.905 (Figure 2).

We investigated also the diagnostic performance by comparing healthy subjects with *in situ* and invasive melanomas, obtaining an AUC of 0.946 (*p* = 0.004) and 0.863 (*p* = 0.004) respectively (Figure 2). In addition we evaluated the prognostic value of the marker by means of ROC curve analysis comparing invasive and metastatic melanomas: the AUC was 0.792 (*p* = 0.021; Figure 2).

### Percentage of tumor DNA in plasma

In order to establish the fraction of tumor DNA circulating in plasma we calculated the percentage of *RASSF1A* methylated DNA versus total cfDNA in samples with detectable levels of the methylated marker. Quantitative data are reported in Table 2.

Healthy subjects have a significantly lower total cfDNA quantity than patients affected by *in situ* (*p* < 0.001), invasive (*p* < 0.001), and metastatic (*p* = 0.001) melanoma.

Patients affected by melanoma show a tendency to have a higher percentage of tumor DNA than controls.

Median values tend to increase from healthy subjects to *in situ*, invasive and metastatic melanomas but the differences among classes are not statistically significant (Figure 1B).

ROC curve analysis revealed a worse diagnostic performance for this relative parameter with respect to the absolute quantification of tumor DNA itself represented by methylated *RASSF1A*. The AUC, in fact, is 0.670 ± 0.105, *p* = 0.155, (95% confidence interval: 0.464-0.877).

No significant correlation was found between methylated *RASSF1A* levels in cfDNA and total cfDNA concentration (Spearman's correlation coefficient ρ = −0.128, *p* = 0.398).

### Comparison between cfDNA and CTCs

With the aim of defining the relationship between CTCs and tumor cell-free DNA, we assessed the presence CTCs in the blood of 68/152 subjects of our case study for whom we could take an additional blood draw. The 68 cases with CTC analyzed were distributed as follows: 12 *in situ* melanomas, 48 invasive melanomas, and 8 metastatic melanomas.

CTCs were detected by an indirect method based on a RT-qPCR assay for tyrosinase mRNA as well as a filtration-based method (ISET).

We found no significant relationship between the presence of CTCs assessed by either method and cfDNA methylated in *RASSF1A* by the Fisher's exact test (Tables 3, 4, *p* = 0.061 and *p* = 0.790, respectively). Analogously, by performing the same analysis in the three different patients' categories of our case study we found no significant association.

**Table 3:** **Distribution of positive and negative cases for *RASSF1A* methylation in cfDNA and for the presence of CTCs as assessed by RT-qPCR for tyrosinase mRNA (Fisher's exact test *p* = 0.061)**.

		**Methylated *RASSF1A***		**Total**
		**neg**	**pos**	
**CTCs (by RT-qPCR for tyrosinase mRNA)**	**neg**	30	20	50
	**pos**	6	12	18
**Total**		36	32	68

**Table 4:** **Distribution of positive and negative cases for *RASSF1A* methylation in cfDNA and for the presence of CTCs as assessed by ISET (Fisher's exact test *p* = 0.790)**.

		**Methylated *RASSF1A***		**Total**
		**neg**	**pos**	
**CTCs (by ISET)**	**neg**	27	23	50
	**pos**	9	9	18
**Total**		36	32	68

On the contrary, the two methods for detecting CTCs showed a significant association between each other by the Fisher's exact test (*p* = 0.013, Table 5). On the other hand, the association was lost when analyzed within patients' categories.

**Table 5:** **Distribution of positive and negative cases for the presence of CTCs as assessed by ISET and RT-qPCR for tyrosinase mRNA (Fisher's exact test *p* = 0.013)**.

		**CTCs (by RT-qPCR for tyrosinase mRNA**		**Total**
		**neg**	**pos**	
**CTCs (by ISET)**	**neg**	41	9	50
	**pos**	9	9	18
**Total**		50	18	68

By combining the determination of the circulating methylated *RASSF1A* marker with the detection of CTCs with either method, we increased the sensitivity of the detection of positive cases in invasive and metastatic melanomas (Table 6).

**Table 6:** **Number of positive cases per class of melanoma patients obtained by assessing *RASSF1A* methylated cfDNA, the presence of CTCs by either method, and by combining the determination of the circulating methylated *RASSF1A* marker with the detection of CTCs**.

	**Number of positive cases/total (%)**		
**Class**	**Methylated *RASSF1A***	**CTC**	**Methylated *RASSF1A* OR CTC**
*In situ*	7/12 (58%)	3/12 (25%)	7/12 (58%)
Invasive	20/48 (42%)	18/48 (37%)	29/48 (60%)
Metastatic	5/8 (62%)	6/8 (75%)	8/8 (100%)

Correlation on the basis of quantitative data between *RASSF1A* and CTC with either method did not provide any significant result (Spearman's correlation, ρ = 0.245, *p* = 0.177 for ISET and ρ = 0.336, *p* = 0.060 for RT-qPCR).

## Discussion

The analysis of cfDNA may have the potential to complement or replace the existing cancer tissue and blood biomarkers in the future (Schwarzenbach et al., 2011). In order to reach this goal, specific and sensitive analytical procedures must be developed and optimized to target circulating molecules showing differences between patients and healthy subjects.

In this study, we investigated the diagnostic performance of *RASSF1A* promoter methylation in cfDNA as a non-invasive marker of tumor DNA in melanoma patients. We focused our attention on the methylated form of the marker since it is supposed to represent the fraction of circulating DNA deriving from the tumor by any of the hypothesized mechanisms of necrosis, apoptosis and active release (Jung et al., 2010).

We considered patients at different stages of melanoma disease in order to test the reliability of the epigenetic marker under study independently from the stage of the tumor. Since the possible sources of CTCs and cfDNA in patients with different clinicopathological characteristics are still non-completely disclosed, we chose to jointly analyze the whole case study in order to test the reliability of the epigenetic marker under study, independently from the stage of the tumor.

Notwithstanding the limited number of cases with a detectable level of methylated *RASSF1A*, especially among healthy subjects, our results demonstrated a good capability of this marker in distinguishing between melanoma patients and healthy control subjects, as evidenced by the ROC curve analysis.

The absolute levels of the epigenetic marker are significantly higher in melanoma compared to controls and increases during tumor progression (from *in situ* to invasive and metastatic disease). In order to evidence both the diagnostic and prognostic potential of the investigated marker, we chose to compare invasive with *in situ* and metastatic melanomas even though the last two patients' categories are less represented in our cohort.

When expressing the results as a percentage of the total amount of cfDNA present in the sample (% tumor DNA) the lack of statistical significance indicates that the tumor DNA values are relevant independently from the cfDNA deriving from different sources within the organism, highlighting that the development of assays that specifically recognize the DNA of tumor origin is a major requirement when analyzing the liquid biopsy.

Moreover, we correlated data on methylated cfDNA with the simultaneous presence of CTCs in the same blood draw of our cohort of subjects in order to investigate the relationship between these two aspects of the liquid biopsy. We found no significant correlation among the two biomarkers analogously to what recently reported in a study on breast cancer patients (Madic et al., 2015).

Some researchers investigated this topic finding an overall agreement between the two parameters (Koyanagi et al., 2006; Schwarzenbach et al., 2009; Van der Auwera et al., 2009; Matuschek et al., 2010; Chimonidou et al., 2013), while others reported that cfDNA is more frequently detected than CTCs in cancer patients (Bidard et al., 2014; Madic et al., 2015). Two studies pointed out a higher sensitivity in mutation detection for tumor cfDNA than CTCs (Punnoose et al., 2012; Freidin et al., 2015). These discrepant results can be partially due to the different methodological approaches used by the researchers. In fact, when comparing these two biomarkers it must be also taken into account that CTC detection is a challenging objective to achieve in the laboratory for the lack of a unique standardized procedure to refer to and for the intrinsic difficulties of the methods for the isolation of these cells which represent a minority of the nucleated blood cells. On the other hand, also cfDNA evaluation is technically challenging due to the low concentration of cfDNA and to the high levels of interfering, non-tumor-specific DNA fragments present in plasma. Moreover, the origin of the cfDNA has not been clarified yet and some authors suggest that tumor cfDNA derives, at least in part, from CTC lysis in the circulation, but nonetheless the two parameters cannot be considered completely overlapping. The most commonly accepted opinion is that CTCs and cfDNA represent two complementary aspect of the liquid biopsy and thus should both be considered to reach a non-invasive approach to cancer management.

In our experience, by the comparison of *RASSF1A* methylated cfDNA with CTCs, it was apparent that they represent two independent biomarkers that could be jointly measured and integrated in the liquid biopsy. The 100% specificity in the detection of metastatic patients was reached by combining the two parameters indicating that both circulating markers should be employed in order to enhance the possibility of disease monitoring in a higher percentage of patients.

The univariate analysis performed so far allowed us to assess the performance of methylated *RASSF1A* as a marker of tumor-related cell-free DNA in melanoma for diagnostic purposes being able to discriminate the *in situ* and invasive melanomas from controls and as an indicator of patients prognosis, as assessed by the good discriminative power of the ROC curve including invasive and metastatic patients.

Further studies are needed to define an algorithm integrating quantitative data related to cfDNA and CTCs.

## Author contributions

FS contributed to the experimental set-up, analysis, interpretation of data and drafting the work; CO contributed to the conception of the work; DM contributed to CTC analysis and critical revision of the manuscript; VD and MG contributed to patients' recruitment, sample collection and clinical data management; MP contributed to the conception of the work and critical revision of the manuscript; PP contributed to the design of the work, interpretation of data, drafting and revising the manuscript. All the authors approved the final version of the manuscript.

### Conflict of interest statement

The authors declare that the research was conducted in the absence of any commercial or financial relationships that could be construed as a potential conflict of interest.
